# Effects of Playing Position and Contextual Factors on Internal Match Loads, Post-Match Recovery and Well-Being Responses of Elite Male Water Polo Players

**DOI:** 10.3390/jfmk8010012

**Published:** 2023-01-18

**Authors:** Andrea Perazzetti, Milivoj Dopsaj, Pierpaolo Sansone, Mauro Mandorino, Antonio Tessitore

**Affiliations:** 1 Faculty of Sport and Physical Education, University of Belgrade, 11000 Belgrade, Serbia; 2 Department of Movement, Human and Health Sciences, University of Rome ‘Foro Italico’, 00135 Rome, Italy; 3 Facultad de Deporte, UCAM Universidad Católica de Murcia, 30107 Murcia, Spain; 4 UCAM Research Center for High Performance Sport, Facultad de Deporte, UCAM Universidad Católica de Murcia, 30830 Murcia, Spain; 5 Performance and Analytics Department, Parma Calcio 1913, 43121 Parma, Italy

**Keywords:** team sport, workload monitoring, RPE, perceived recovery, Hooper’s Index

## Abstract

This study aimed to investigate the effects of playing position and contextual factors (match outcome, final score difference, match location, travel duration, number of scored and conceded goals) on the internal match load, players’ perceived recovery and players’ well-being. The session-RPE (s-RPE), Perceived Recovery Scale (PRS) and Hooper Index (HI) of 17 male elite water polo players were monitored during all matches (regular season and play-out) of the 2021/22 Italian Serie A1 championship. Three separate, mixed linear models for repeated measures showed significant main effects: drawn compared to won matches led to higher s-RPE values (mean ± SE = 277 ± 17.6 vs. 237.3 ± 20.6), while longer travel duration (estimate = −0.148) and goals scored (estimate = −3.598) led to lower s-RPE values; balanced compared to unbalanced matches led to higher PRS values (mean ± SE = 6.8 ± 0.3 vs. 5.1 ± 0.4), while playing time (estimate = −0.041) and goals scored (estimate = −0.180) led to lower PRS values; higher scores of the HI were registered for regular season compared to the play-out (mean ± SE = 15.6 ± 0.9 vs. 13.5 ± 0.8). This study marks the importance of ecological and non-invasive monitoring tools to assess internal match load, recovery and the well-being of elite water polo players.

## 1. Introduction

Water polo is an intermittent, high-intensity, body-contact aquatic team sport. Since the end of the nineteenth century, its five historical developmental stages [1] have seen several changes of the play’s rules, with an incessant increase in game demands [2]. Consequently, water polo players are required to have higher conditioning levels and more advanced technical and tactical skills. The intermittent nature of the game requires the concurrent contribution of anaerobic and aerobic energy metabolism [3]. The former supports the high-intensity and short-duration activity, while the latter sustains the low to moderate intensity of longer actions [4,5].

Previous studies on water polo’s game demands have shown that two thirds of the playing time is spent above 85% of the peak heart rate (HRpeak), which (approximately) corresponds to the lactate threshold intensity [6]. Therefore, performing high-intensity activities with short recovery periods is crucial, and high strength and power levels are prerequisites for high-standard players [4,7,8]. Moreover, the current high-level competition system requires water polo players to perform multiple demanding training sessions and games throughout the season, from the high-competitive friendly matches of the pre-season to the official ones of the in-season [9]. For this reason, it is essential to carefully manage the players’ training and competition loads, allowing the necessary recovery to optimize performance and avoid injury and illness [10,11,12]. 

Monitoring players’ training loads, recovery and well-being is becoming a common practice in high-level water polo [8]. A strategic priority for successful coaching (i.e., focused on optimal training) is to monitor players’ psychophysiological internal load responses to cope with the demands elicited by the external loads in both training and competitions [13,14,15], during the different phases of the season [9]. 

According to the relevant literature, there is not a single or gold standard method to measure the external and internal loads [16]. Although the importance of using other subjective measures cannot be underestimated, one of the most used methods to assess the internal load is through the rating of perceived exertion (RPE) [10,17]. Based on the RPE measure, Foster et al. [18] developed the session-RPE method, which considers the intensity and duration of the training/competition sessions to calculate the training and competition loads [19]. Over the years, this method has been validated to monitor the load of different training modalities (technical, tactical, endurance, speed and strength) across multiple sports, during one or more sessions or week(s) [20,21], including water polo [22,23]. In particular, the study by Lupo et al. [22] that compared the Edwards’ heart-rate-based method and the session-RPE method to assess the players’ internal load during trainings of a U17 team competing at national level, showed that the s-RPE was a reliable method to evaluate the internal training load (ITL) in youth water polo. The study by Botonis et al. [23] used the s-RPE method to assess the ITL during two short-duration (2 weeks each) training periods (overloaded vs. reduced training) of a Greek First League water polo team. However, there is a lack of studies to monitor and assess the ITL after the official matches of a First League water polo team during an entire water polo season.

Alongside training load, it is essential to control and optimize players’ recovery status to design appropriate training plans that finally leads to positive training adaptations [24,25]. In this regard, Kenttä and Hassmén [26] introduced the use of the Total Quality Recovery Scale (TQR), like the ordinary Borg 6-20 RPE scale, which allows the evaluation of a player’s recovery status. Successively, Laurent et al. [27] introduced a Perceived Recovery Scale based on a 0–10 scalar representation of the individual’s level of perceived recovery. Currently, players’ recovery in water polo has been investigated only regarding nutritional strategies, ergogenic supplementation and heart rate variability, while there is limited research that uses the Perceived Recovery scale [8]. Moreover, to the authors’ knowledge, there are no studies that investigate the players’ perceived recovery after the match during an entire elite water polo season. 

Another perceptual wellness measure that has grown in popularity, has been introduced by Hooper and Mackinnon [28]. This questionnaire rates the well-being considering indexes of fatigue, stress levels, delayed onset muscle soreness (DOMS) and sleep quality/disorders. The Hooper questionnaire has been widely implemented by team sport researchers, with significant associations found between training loads and the Hooper Index (HI) [29,30]. Previous studies on water polo [23], with the addition of the mood measure, showed that players’ overall wellness scores were highly correlated to their internal training loads during a training tapering phase before a competition. Altogether, the HI appears sensitive to changes in training loads, and is therefore a potentially useful monitoring tool; however, it has been scarcely implemented in water polo research.

In a team sport invasion game (e.g., water polo), in addition to the physical, technical and mental level of preparedness, the players’ match load can be influenced by some contextual factors, and interaction has been progressively studied in recent years [31]. These factors include both variables related to the game schedule, such as game frequency and game location (home or away), and variables related to the level of opposition between the two teams, such as match outcome, match status and score-line. Moreover, sport enjoyment has been defined as ‘a positive affective response to the sport experience that reflects feelings and/or perceptions such as pleasure, liking, and experienced fun’ [32]. To the authors’ knowledge, there is still limited information about how playing positions and contextual factors impact on the players’ internal match load, perceived recovery and well-being during an entire water polo season. Therefore, the aim of this study was to investigate the independent effects of the playing position and match-specific contextual factors on the internal match load, the post-match status of perceived recovery and the well-being of players after official elite water polo matches.

## 2. Materials and Methods

### 2.1. Experimental Design

This study used an observational longitudinal design to monitor and assess the perception of internal match loads, perceived recovery and well-being in male elite water polo players during 19 official matches (regular phase, n = 13; play-out phase, n = 6) of the 2021/22 water polo season. During these competitive weeks, the team carried out the following weekly microcycle: 3 team-based water polo sessions (250 ± 47 min per week) focused on technical skills and game-based conditioning; 2 physical training sessions (139 ± 30 min per week and 3842 ± 905 m of conventional swim per week, and 133 ± 27 min per week of resistance training in the gym); 2 friendly matches (142 ± 68 min per week); 1 official match (53 ± 3.5 min per match) and 1 day of rest.

To calculate the match internal load through the s-RPE method [19,33], players’ RPE values were obtained by means of the Italian translation of the Borg category-ratio 10 scale (CR-10) [34], modified by Foster et al. [18], asking each player ’How intense was your session?’ at the end of each official match. Then, players’ perceived recovery and well-being were collected 36 h post-match by means of the Perceived Recovery Scale (PRS) [27] and Hooper Index (HI) questionnaire [28], respectively, before the beginning of the first weekly training session [35]. To avoid potential differences in individual recovery conditions, players were asked to abstain from recovery interventions and invited to maintain their usual lifestyle for the 36 h prior to the monitoring of PRS and HI.

Then, players’ internal match load, perceived recovery and well-being were analyzed in relation to the following contextual factors: (a) playing time, defined as the player’s total minutes of play in each official match, which also included the player exclusion during a ’man-down’ situation; (b) match outcome, defined as the team’s winning, drawing and losing result at the end of the match; (c) final score difference, defined as the difference of goals scored between the two teams in a match (balanced: ≤3 goals vs. unbalanced: >3 goals) [36]; (d) match location (home or away); (e) travel duration, measured in minutes of travel for each match played away; (f) number of goals scored and goals conceded by the observed team in each match; and (g) season phase: based on the Italian water polo First League, which divided the 2021/22 championship schedule into regular season (n = 13 matches) and play-out (n = 6 matches). 

### 2.2. Subjects

Seventeen male water polo players (mean ± SD, age: 25.9 ± 6.2 years; height: 185.8 ± 8.3 cm; body mass: 87.6 ± 9.9 kg; total playing experience: 15.1 ± 5.4 years; experience in first teams: 9.1 ± 6.1 years; experience in first division: >5 years), belonging to the first team roster of the ’S.S. Lazio Nuoto’ club, which competed in the Italian First League water polo championship (Serie A1), participated in this study. Before the commencement of data collection, all subjects signed a written informed consent form, and the study design was approved by the local research ethics committee of the University of Rome ’Foro Italico’ (CAR 99/2021). To classify their activity level and athletic ability, all players were classified as tier 3 ‘Highly Trained/National Level’, according to the ‘Participant Classification Framework’ of McKay et al. [37]. All subjects regularly engaged in training and competitions during the season and were classified according to their principal playing position [38]: perimetral (or peripheral) players (n = 10); center defenders (n = 3); center forwards (n = 2) and goalkeepers (n = 2). 

Furthermore, the following subject inclusion criteria were used for the final analysis: (a) only data from players employed for at least the mean total duration of a quarter (15 min) of playing time in a match were considered; (b) not presenting any injury or illness that could impair player’s performance; and (c) the goalkeepers were excluded from the sample.

### 2.3. Procedures

Regarding the anthropometric features, players’ stature was measured using a SECA 213 Stadiometer (measuring range 20–205 cm, SECA, Hamburg, Germany), while body mass was obtained using a Tanita SECA 762 (measuring range 0–150 kg, SECA, Hamburg, Germany) to assess the players’ BMI. Age, years of experience and playing role were also registered.

Players’ RPE values were collected about 30 min after the end of the match. To avoid potential interferences of the post-match environment and synchronize the timing of their answers, players were familiarized to use a phone-based online application (proved to be a valid tool in elite sport [39]), by answering the RPE scale using a customized google forms (www.docs.google.com/forms accessed on 12 September 2021) questionnaire to replicate the printed scale content and formatting, which was sent via WhatsApp on their personal smartphone. The internal match load was then calculated with the s-RPE method [19] by multiplying the players’ perceived exertion values by the time (in minutes) played into the match. The s-RPE values are expressed in arbitrary units (a.u.). This method has been shown to be a valid method for monitoring water polo players [22,23]. 

To individually assess perceived recovery and well-being status, players reported their answers to the same researcher after seeing and filling printed scales every Monday morning prior to the training session. The perceived recovery was assessed using a modified version of the TQR scale [40] by means of a 10-point Perceived Recovery Scale [27], ranging from 0 (’very poorly recovered/Extremely tired’) to 10 (’Very well recorded/Highly energetic’), which has already been used in team sports [41,42] and has shown itself to be a valid tool for water polo [8]. The players’ well-being was monitored through the Hooper Index [28], by means of a 7-point Likert scale for the four Hooper Scale categories; (i) stress; (ii) fatigue; (iii) muscle soreness (DOMS); and (iv) sleep. The subset Likert scales ranged from 1 (‘very, very low‘) to 7 (‘very, very high’) for stress, fatigue and DOMS, and from 1 (’very, very bad’) to 7 (’very, very good’) for sleep, respectively. Then, the overall Hooper Index of well-being was provided by summating the four subjective ratings. Finally, the water polo players rated their overall enjoyment of the match activity on a 7-point Likert scale (1 = not at all, 7 = extremely enjoyed). 

Before the commencement of the study, during the first three weeks of pre-season, all players were familiarized with RPE, PRS, HI and enjoyment scales. To individually create awareness of the possible responses, each player received a printed copy of the different questionnaires and was instructed to read and interpret the scales before and after each training session and friendly match.

### 2.4. Statistical Analysis

Three separate mixed linear models for repeated measures (matches) were performed to evaluate the single main effects of contextual factors on three dependent variables: s-RPE, PRS and HI. The factors included in the three mixed linear models are presented in Table 1. The player was included as a random factor [43]. Assumption of normality for the variables included in the models was evaluated by residuals plots, which were normally distributed [44]. A second mixed linear model was conducted for interaction effects for each dependent variable, considering only those factors that showed a significant main effect in the first model. Statistical analyses were performed using SPSS version 26.0 (IBM, Chicago, IL, USA). For each variable, descriptive data are expressed as mean ± SD. Significance was set at *p* ≤ 0.05. Results of the main effect analysis are reported as F and *p* values, while the estimate value is reported for the statistically significant factors. Post-hoc pairwise comparisons were assessed using the Bonferroni test. Effect sizes (ES) for pairwise comparisons were calculated using Cohen’s d, with the following interpretation: 0.2, trivial; 0.2–0.6, small; 0.6–1.2, moderate; 1.2–2.0, large; and 2.0, very large [45]. Results of the pairwise comparisons are described in mean ± standard error (SE), *p*-value, mean difference and ES.

## 3. Results

Descriptive data (mean ± SD) of s-RPE, PRS and HI are presented in Table 2. 

During the study period, the team played 19 official matches, scoring an average of 8 ± 3 goalsand conceding 14 ± 5 goals per match, while a total of 22 ± 4 goals were scored by the two teams per match. Moreover, during the season the team played 9 away matches, with an average travel duration of 218 ± 125 minutes. 

**Figure 1:**
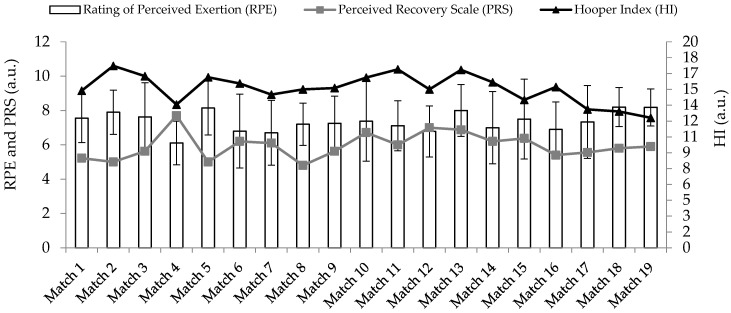
Trends of RPE, PRS and HI scores across the season.

Regarding perceptual responses, the mean value of each variable was: RPE = 7.3 ± 0.6 a.u.; enjoyment = 3.8 ± 1.1 a.u.; sleep quality = 3.5 ± 0.4 a.u.; stress = 4.1 ± 0.5 a.u.; fatigue = 4.2 ± 0.4 a.u.; DOMS = 3.5± 0.6 a.u.; and PRS = 5.9 ± 1.7 a.u.. Figure 1 shows the trends of RPE, PRS and HI scores across the season.

Table 3 presents the significant main effects found. For s-RPE, the pairwise comparisons showed significative effects in match outcomes (*p* ≤ 0.05), showing that drawn matches led to higher s-RPE (mean ± SE = 277 ± 17.6 a.u.) than won matches (237.3 ± 20.6 a.u.) (mean difference = 39.7 ± 12.4 a.u.; *p* = 0.015; ES = 1.46, large). A significant effect of travel duration was found on s-RPE (estimate = −0.148), demonstrating how longer travel duration decreased the s-RPE value. For playing time (estimate = 9.328), it was shown that higher playing times led to higher s-RPE scores, while the higher number of goals scored (estimate = −3.598) led to a lower s-RPE score. No significant effect was found for playing position, season phase, match location, final score difference or goals conceded. Regarding PRS, a significative main effect was found for the final score difference (*p* ≤ 0.05), with pairwise analysis showing that PRS was higher after balanced matches (mean ± SE = 6.8 ± 0.3 a.u.) compared to unbalanced matches (5.1 ± 0.4) (mean difference = 1.669 ± 0.362; *p* < 0.001, ES = 1.57, large). Significant effects for PRS were also found for playing time (estimate = −0.041), for goals scored (estimate = −0.180) and for perceived enjoyment (estimate = −0.114), showing that higher levels of these factors decreased PRS scores. Differently, goals conceded (estimate = 0.138) had a positive impact on PRS. In this case, no significant effects were found for playing position, season phase, match location, match outcome, travel duration and RPE. For HI, significant effects were found for the season phase and match outcome (all *p* ≤ 0.05). Specifically, post-hoc pairwise comparisons demonstrated higher scores of HI in the regular season (mean ± SE = 15.6 ± 0.9 a.u.) compared to the play-out phase (13.5 ± 0.8 a.u.) (mean difference = 2.1 ± 0.5 a.u.; *p* < 0.001, ES = 0.78, moderate), as well as in won matches (mean ± SE = 16.1 ± 1.1 a.u.) compared to lost matches (13.9 ± 0.8 a.u.) (mean difference = 2.3 ± 0.9 a.u.; *p* = 0.049, ES = 0.90, moderate), and won matches in comparison with drawn matches (13.6 ± 1 a.u.) (mean difference = 2.5 ± 0.8 a.u.; *p* = 0.009, ES = 1.68, large), respectively. Furthermore, significant effects for HI were found for RPE (estimate = 0.266), showing that a higher value of match RPE led to a higher HI score at the beginning of the week. No significant effects were found for playing position, match location, final score difference, travel duration, playing time, goals scored, goals conceded and perceived enjoyment.

Regarding the interaction effects, no interactions were found for s-RPE, while PRS was influenced by the combination of playing time and final score difference (F = 11.686; *p* < 0.001; balanced, estimate = −0.106; unbalanced, estimate = −0.098), and by the playing time and perceived enjoyment (F = 7.980; *p* = 0.006; estimate = 0.017). The first interaction had a negative impact on the PRS, decreasing the value of this variable in both conditions of the final score difference. On the contrary, the second interaction showed that higher playing time in combination with higher perceived enjoyment led to a higher value of PRS. Regarding HI, one interaction was found, namely between season phase and RPE (F = 5.519; *p* = 0.024; regular season, estimate = −0.055; play-out, estimate = −0.668), suggesting that these factors decreased the value of HI score, especially in the play-out season.

## 4. Discussion

This study is the first to monitor First League water polo players after official matches during an entire water polo season (regular and play-out phases), to investigate the independent effects of the playing position and match-specific contextual factors on the perceived internal match load, post-match perceived recovery status and players’ well-being.

The first analysis of this study concerned the potential influence of the playing position, playing time, match outcome, final score difference, travel duration, match location, goals scored and goals conceded on match s-RPE. Among these factors, the results showed that only the playing time, match outcome, goals scored, and travel duration played a significant role on the players’ match s-RPE. 

Previous studies on water polo showed significant differences in the players’ physical fitness levels and match external loads [3,38,46,47,48] in relation to their different playing roles. Although not significant, our study confirmed this trend for the s-RPE, with higher mean s-RPE values experienced by perimetral players compared to center defenders and center forwards. Such differences can be explained by the different technical and tactical tasks performed by each water polo role, with perimetral players covering a higher total swim distance than center forwards [49] and engaging in longer and more frequent high-intensity swimming than center-defenders [50,51]. In fact, compared to center positions (defenders and forwards), the perimetral players are more involved in both defensive and offensive phases of play (e.g., number of counterattacks), which require repeated high-intensity swimming to quickly move between the two sides (i.e., defensive and offensive) of the field [52]. This condition could explain why we have higher match s-RPE trend for this playing position. 

Regarding the effect of playing time on players’ s-RPE, our data showed a significant effect for playing time. Although both the playing time (in minutes) and RPE value contributed to the arbitrary unit amount of the s-RPE (i.e., minutes × RPE), the effect of playing time can be explained by the fact that there were between-role differences, with perimetral players registering the highest amount of total playing time compared to the other roles.

Another contextual factor analyzed in our study has been the match outcome, which showed a significant effect on players’ s-RPE, with higher mean values registered after drawn matches compared to won and lost ones. Although it could be expected to register higher values as a consequence of a lost match, during the season the team lost 15 matches. In this regard, Oliveira et al. [53] warn that in team sports matches where the opposition of the two teams is unbalanced since the first quarter of the match [5], the intensity of the dispute could lose significance and even determine a lower perception of fatigue. In fact, during drawn matches, both teams remain potential winners until the end of the match, requiring a greater effort from the players in the attempt to subvert the result until the last moment. This explanation might also explain our findings for the scored and conceded goals. In fact, to maintain a balanced status until the end of the match, a team should provide the maximal effort to score goals, while conceding the minimum ones [54]. In this regard, our findings showed that a higher number of goals scored led to a higher match s-RPE, while the goals conceded did not influence the match internal load of our subjects. This could be explained by the fact that in our study, the team registered an unbalanced final score (e.g., conceding a lot of goals) in two/thirds of the monitored matches.

In team sports, the game location has been identified as influencing the performance [55]. In water polo, although the exact causes are still not clear, Ruano et al. [5] demonstrated that the match location influenced the match outcome, with negative effects on the teams playing away. In our study, this contextual factor did not influence the players’ s-RPE, while the travel duration showed to be significant. However, contrarily to what could be expected, our data showed that the longer the travel duration, the lower the players’ s-RPE. Once again, as we stated above, an explanation of this contradictory result regards the intensity of the two teams in opposition in the match. In fact, of the nine matches played away, our team lost eight matches, most of which after a longer travel (291 ± 95 min), with an unbalanced final score difference and a higher number of goals conceded, which could have determined an even lower perception of fatigue due to this lower ’duel’ intensity.

The other main aims of this study concerned the players’ perceived recovery and well-being statuses, and to investigate how these two dependent variables were influenced by match contextual factors and demands. Among these factors, the results showed that the players’ post-match recovery was influenced by the final score difference, playing time, perceived enjoyment, goals scored, goals conceded, season phase, match outcome and match RPE.

The analysis of s-RPE showed a significant effect only on the match outcome, while the final score difference did not show a significant influence. However, the latter had a significant effect on players’ perceptions of recovery 36 h post-match, showing higher PRS mean values registered after balanced matches compared to unbalanced ones. This result could be explained by the fact that our players considered balanced matches more gratifying than the unbalanced ones, which might have had an impact on their mood and/or enjoyment. [56]. Such consideration can be further supported by the higher values of players’ perceived level of enjoyment, which registered higher values after balanced matches (AU = 4.2 ± 1.4) compared to unbalanced ones (AU = 3.2 ± 1.3). Investigating the technical and tactical aspects of elite water polo matches in relation to margins of victory, Lupo et al. [36] showed that in balanced matches, a higher number of opponent’s exclusions fouls performed led to more power-play actions, which might contribute to bestowing greater match enjoyment in players. 

The players’ level of perceived recovery has also been influenced by their amount of time spent in the water during the match, with a higher playing time corresponding to lower perceived recovery scores (the higher the volume of play, the lower the quality of recovery). Additionally, this result could have been expected by the fact that, although not significant, the RPE showed a trend for a negative effect (estimate = −0.114, *p* = 0.055). Our findings are in line with previous water polo research, showing how reducing training load by 30% led to significant improvements in perceived recovery status [57]. Therefore, our study poses the focus on the fact that in a standard microcycle, the status of perceived recovery at the beginning of the week is strongly influenced by the last competition [58].

Moreover, proving that some contextual factors are intertwined, we observed that increasing the number of team goals scored corresponded to a lower score of perceived recovery, which is in line with the higher s-RPE values registered for this parameter. On the contrary, in line with our previous statement that the goals conceded did not influence the match internal load, after matches lost with a higher number of conceded goals our players registered a higher score of perceived recovery. Although, this aspect might appear as a contradiction, a technical explanation of this fact is that a clear, unbalanced level between the two opponent teams (which could be related with a high number of goals conceded) leads to a lower frequency of consecutive actions, a higher number of breaks after a goal and consequently, a more frequent use of player substitutions [59]. In fact, in water polo, there are several occasions when a game is stopped, which allows coaches and players to modify their strategy and tactical behavior several times during the match [60].

Moreover, the analysis of players’ well-being was also in line with the previous statements for s-RPE and perceived recovery. In fact, we found a general tendency (non-significant, *p* = 0.083) to observe higher perceived enjoyment values in relation to a lower HI total score (higher well-being). This fact can be confirmed by the higher HI scores observed during the regular season, when the team lost twelve matches and drew one (in thirteen matches), with the higher number of unbalanced matches. 

## 5. Limitations and Future Research Perspectives

This study has some limitations that warrant discussion. The RPE (then the derived s-RPE), PRS, HI and enjoyment were only monitored for official matches, while no data were collected during the weekly training sessions. Though we have summarized the team’s typical weekly microcycle, a more detailed external loads monitoring that also includes a time–motion analysis of game-based conditioning and small-sided-games, could improve the results interpretation. Based on these limitations, future studies could investigate the correlation between the variables used in this work with weekly external loads data. Furthermore, we did not consider the occurrence of technical and tactical events, which also determine the frequency of players’ direct involvement with the play and could influence both the players’ fatigue and enjoyment. For this reason, in further studies, both the time–motion and notational analysis data could be additionally collected.

## 6. Conclusions

Although this study has limitations, it has provided a new strategy to monitor water polo players since, as far as the authors’ knowledge, this is the first work investigating the effects of different contextual factors on s-RPE, PRS and HI values during an entire competitive season in elite water polo. In fact, the findings of this study show the importance of monitoring the s-RPE, post-match recovery and well-being status of water polo players on a weekly basis. In particular, the monitoring of the players’ perceived psychophysiological status during the ‘critical’ days of transition between the last match (i.e., last day of the previous microcycle) and the first training session (i.e., first day of the new microcycle) could help coaching staff to organize accurate and tailored training sessions according to the contextual factors and the overall status perceived by their players. 

However, the findings of our study also show that perceived internal load, recovery, and well-being should not be used interchangeably. For this reason, as a practical strategy, the coaching staff are advised to monitor all these parameters since specific contextual factors could determine different responses. In fact, in a match, the intensity of the ‘dispute’ (opposition) between two teams, can mediate the RPE (and then the s-RPE). For instance, as demonstrated in our study, losing a match with a larger final score difference (unbalanced) determined a lower s-RPE compared to matches that were more balanced (e.g., drawn matches).

## Figures and Tables

**Table 1:** Factors included in the main effect analysis.

Session-RPE	Perceived Recovery Scale (PRS)	Hooper Index (HI)
Playing position	Playing position	Playing position
Playing time	Playing time	Playing time
Season phase	Season phase	Season phase
Match location	Match location	Match location
Match outcome	Match outcome	Match outcome
Final score difference	Final score difference	Final score difference
Travel duration	Travel duration	Travel duration
Goals scored	Goals scored	Goals scored
Goals conceded	Goals conceded	Goals conceded
	RPE	RPE
	Enjoyment	Enjoyment

**Table 2:** Descriptive data (mean ± SD) of the contextual factors.

Contextual Factors	Session-RPE (a.u.)	Perceived Recovery Scale (PRS) (a.u.)	Hooper Index (HI) (a.u.)
Playing position			
perimetral players (n = 10)	296.2 ± 102.7	5.5 ± 1.8	16.1 ± 3.7
center defenders (n = 3)	187.6 ± 62.2	6.3 ± 1.4	14.8 ± 3.3
center forwards (n = 2)	175.9 ± 72.4	6.4 ± 1.4	12.9 ± 2.7
goalkeepers * (n = 2)	293.1 ± 143.4	6.4 ± 2.1	20.2 ± 2.6
Season phase			
all-season (n = 19)	246.4 ± 104.9	5.9 ± 1.7	15.2 ± 3.6
regular season (n = 13)	246.8 ± 103.4	5.9 ± 1.6	15.5 ± 3.7
play-out (n = 6)	245.8 ± 108.4	5.9 ± 1.7	14.55 ± 3.5
Match location			
home (n = 10)	257.4 ± 113.1	5.8 ± 1.7	15.4 ± 3.9
away (n = 9)	232.3 ± 92	6.1 ± 1.6	14.7 ± 1.6
Match outcome			
win (n = 2)	264.1 ± 120.4	5.9 ± 2.2	15.5 ± 3.7
draw (n = 2)	258.6 ± 118.6	5.6 ± 1.1	14.5 ± 3.8
loss (n = 15)	242.6 ± 101.4	6 ± 1.7	15.2 ± 3.6
Final score difference			
balanced (n = 10)	261.1 ± 117.8	6 ± 1.7	15.3 ± 3.7
unbalanced (n = 9)	231.1 ± 3.5	5.9 ± 1.6	15 ± 3.5

* Goalkeepers were only included in the descriptive statistic of playing position.

**Table 3:** Results of the main effect analysis *.

Session-RPE	F	*p*	Perceived Recovery Scale (PRS)	F	*p*	Hooper Index (HI)	F	*p*
playing position	0.106	0.900	playing position	0.185	0.833	playing position	0.781	0.480
playing time	1173.321	**<0.001**	playing time	16.702	**0.001**	playing time	0.582	0.449
season phase	0.146	0.706	season phase	0.016	0.898	season phase	16.673	**<0.001**
match location	3.424	0.076	match location	0.217	0.645	match location	0.439	0.515
match outcome	7.176	**0.003**	match outcome	0.428	0.656	match outcome	5.447	**0.008**
final score difference	1.147	0.290	final score difference	21.258	**<0.001**	final score difference	3.849	0.059
travel duration	13.873	**<0.001**	travel duration	0.001	0.975	travel duration	1.148	0.313
goals scored	4.145	**0.050**	goals scored	9.920	**0.003**	goals scored	3.879	0.054
goals conceded	0.246	0.622	goals conceded	15.263	**<0.001**	goals conceded	0.007	0.934
			RPE	4.181	0.055	RPE	5.225	0.026
			enjoyment	6.670	0.016	enjoyment	3.086	0.083

* Significant effects in bold.

## Data Availability

Not applicable.
